# Impact of National Centralized Drug Procurement policy on chemical pharmaceutical enterprises’ R&D investment: a difference-in-differences analysis in China

**DOI:** 10.3389/fpubh.2024.1402581

**Published:** 2024-07-01

**Authors:** Jiaming Li, Xinyue Zhang, Rui Wang, Keyao Cao, Luhui Wan, Xu Ren, Jinxi Ding, Wei Li

**Affiliations:** ^1^School of International Pharmaceutical Business, China Pharmaceutical University, Nanjing, China; ^2^The Second Affiliated Hospital of Zhejiang Chinese Medical University, Xinhua Hospital of Zhejiang Province, Hangzhou, China; ^3^Pharmaceutical Market Access Policy Research Center, China Pharmaceutical University, Nanjing, China

**Keywords:** National Centralized Drug Procurement policy, chemical pharmaceutical enterprise, R&D investment, influence mechanism, difference-in-difference analysis

## Abstract

**Objective:**

This study aimed to evaluate the impact of the National Centralized Drug Procurement (NCDP) policy on chemical pharmaceutical enterprises’ R&D investment and provide references for improving NCDP policy design and encouraging innovation in the pharmaceutical industry.

**Methods:**

Using the panel data of 102 Shanghai and Shenzhen A-share listed enterprises from 2016 to 2022 under the chemical pharmaceutical classification of Shenwan in Wind database as the research sample, this study developed difference-in-differences (DID) models on bid-winning and bid-non-winning enterprises, respectively, to evaluate the impact of NCDP policy on their R&D investment. In addition, this study tested the heterogeneity of bid-winning enterprises based on the bid success rate, the decline of drug price, and enterprise size.

**Results:**

The NCDP policy could encourage chemical pharmaceutical companies to increase R&D investment, but the low bid success rate and excessive drug price reduction would reduce their R&D enthusiasm, especially for small- and medium-sized enterprises.

**Discussion:**

It is suggested that the NCDP policy should be further improved: first, revise the bidding rule of the NCDP policy and increase the bid success rate so that more enterprises can win bids, and second, to solve the problem of excessive drug price reduction, evaluate the rationality of bid-winning prices, and introduce a two-way selection mechanism between medical institutions and supply enterprises. Integrate pharmacoeconomic evaluation into the NCDP rules to form a benign competition among enterprises. Third, attention should be paid to supporting policies for small- and medium-sized enterprises. By increasing procurement volume, shortening payment time limits, and increasing the proportion of advance payments, enterprises’ cash flow shortages can be alleviated, thus achieving fairness and inclusiveness in the implementation of the NCDP policy.

## 1 Introduction

With the overall improvement of global pharmaceutical R&D technology, drug expenditures have been increasing rapidly, greatly increasing patients’ financial burden (1–3). Therefore, the rationality of the growth rate of drug costs has become a hot topic for health and medical insurance departments worldwide. At present, implementing government-lead bidding and procurement system for drugs has become one of the main cost control measures around the world (4–7).

To ease the burden on patients, the Chinese government launched a centralized drug bidding and procurement system at the provincial level in 2009 (8), whereby a drug procurement platform was established under the unified leadership and supervision of provincial governments. However, due to the limitation that the bid-winning price was determined without guaranteeing procurement quantity, and the contract did not stipulate requirements for procurement quantity and payment time, enterprises lacked sales volume expectations and payment guarantees. Therefore, such a procurement pattern of “only bidding, no procurement” neither reduced enterprises’ selling costs nor created a good market competition environment (9). Problems such as high drug prices and kickbacks kept emerging in endlessly. In particular, the prices of off-patent drugs remained high, and the “Patent Cliff” phenomenon failed to form (10–12).

In this context, pharmaceutical expenditures in China continued to grow at an average annual rate of 14% (13), accounting for 30 to 40% of total healthcare expenses from 2010 to 2018 (14). To lower the price of off-patent drugs and alleviate the financial burden on patients, in December 2018, the Chinese government implemented the National Centralized Drug Procurement (NCDP) policy (15). The initial pilot of NCDP was implemented in four municipalities (Beijing, Tianjin, Shanghai, and Chongqing) and seven sub-provincial cities (Shenyang, Dalian, Xiamen, Guangzhou, Shenzhen, Chengdu, and Xi’an) in Mainland China, hence referred to as the “4 + 7” pilot. Starting in 2019, the pilot expanded nationwide. A key difference between NCDP and previous drug procurements is that traditional drug procurements were conducted at the regional level, with each province responsible for its drug procurement without cross-procurement with other provinces. The NCDP operates at the national level, allowing for better organization and coordination among medical insurance, medical institutions, and pharmaceutical companies. This enables comprehensive and systematic implementation of overall planning and procurement.

Different from traditional centralized procurement, the NCDP policy stipulates the need to specify the procurement volume during the bidding process. For off-patent drugs with multiple generic alternatives, the government is responsible for organizing the bidding, and medical institutions report their demand and expected procurement ratios in advance to determine the procurement quantity. The bidding is open to all pharmaceutical companies in China, and companies compete based on the “exchange volume for price” principle through competitive negotiations. Brand-name drugs and generic drugs that have passed consistency evaluations compete on the same platform. The enterprise offering the lowest price wins the bid and obtains 50% ~ 80% market share (16). In the initial pilot round, exclusive bidding was awarded to the bidder that offered the lowest price, gradually transitioning to a mechanism of “multiple winners.” Market share allocation adopts the “sequential allocation” method, where enterprises offering the lowest price can select one supply region first. After their selection, other enterprises then choose supply regions in order of price from low to high. Therefore, the lower the price, the more the market share, thereby encouraging companies to lower drug prices. Additionally, the NCDP policy requires medical institutions to prioritize the use of drugs from bid-winning companies, fulfill the agreed purchase quantities, and repay on time, which can effectively alleviate the burden on enterprises. As of December 2023, the Chinese government has carried out nine rounds of NCDP, mainly focusing on chemical drugs, including 374 off-patent drugs involving fields such as diabetes and hypertension, with an average price reduction of more than 50%.

It can be seen that chemical pharmaceutical enterprises are the main participants in the NCDP. Whether the NCDP policy can maintain the long-term sound operation and innovative development of chemical pharmaceutical enterprises is the prerequisite for maintaining drug quality, quantity, and stable supply (17). Therefore, evaluating the impact of the NCDP policy on enterprises’ R&D investment is of profound meaning. However, the implementation of the NCDP policy has led to a sharp decline in drug prices, resulting in the shrinkage of the off-patent drug market. In a short period, the overall profits of chemical pharmaceutical enterprises participating in the NCDP have decreased (18). Whether chemical pharmaceutical enterprises could adapt to the new procurement environment and whether they have sufficient resources to promote sustainable innovative development remain to be verified.

Current research on the NCDP policy mainly focuses on drugs, examining the policy effects on drug expenses and usage. However, there is scarce literature that explores the impact of the NCDP policy on enterprises’ R&D investment from the perspective of stakeholders. Most of previous research focus on theoretical analysis (19–21) and lack empirical studies (22, 23). From the theoretical perspective, scholars have conflicting views. Some believe that the NCDP policy has reduced drug prices sharply, which has negative impacts on the profitability of chemical pharmaceutical enterprises. In addition, the R&D costs of innovative drugs are huge; therefore, enterprises usually take cost-saving measures and reduce their spending on R&D (24–26). Others believe that the NCDP policy contributes to reasonable competition in the pharmaceutical industry (27, 28). Faced with the challenges of sustainable business operation and cost control, enterprises would pay more attention to R&D and take the initiative to transform themselves into innovative and technology-based enterprises (29, 30). The main reason behind this divergence lies in the uncertainty of whether the enterprises’ profit could cover the spending on R&D activities. Winning bids and reducing the prices of bid-winning drugs directly impact future profits, thereby affecting their future development strategy and R&D investment. However, little research has conducted empirical analysis regarding the impact of the NCDP policy on bid-winning and bid-non-winning enterprises and lacks targeted research from perspectives of the bid-winning rate, changes in price reduction, and so on.

To sum up, from an empirical perspective, this study used a difference-in-differences (DID) model to evaluate the impact of the NCDP policy on the R&D investment of bid-winning and bid-non-winning chemical pharmaceutical enterprises. In addition, the relationship between the NCDP policy and R&D investment was tested from the perspective of heterogeneity, including bid success rate, drug price reduction, and enterprise size. Therefore, our study provides valuable insights into the transformation of chemical pharmaceutical enterprises and the reform of the pharmaceutical industry.

## 2 Theory and hypothesis

For chemical pharmaceutical enterprises, the most significant difference in the impact of the NCDP policy is whether their drugs win the bid. Previous research mostly analyzes all chemical pharmaceutical enterprises in a general way and lacks comparative analysis between bid-winning and bid-non-winning enterprises, resulting in conflicting views of different scholars. Given the inherent mechanism, bid-winning enterprises obtain more market share, while bid-non-winning enterprises may face the problems, such as shrinking market share and declining sales, which would directly affect profits (31). Therefore, there may be differences in the R&D decisions between the two, and differential analysis is required.

### 2.1 Theoretical analysis

#### 2.1.1 Mechanism of influence

**Figure 1:**
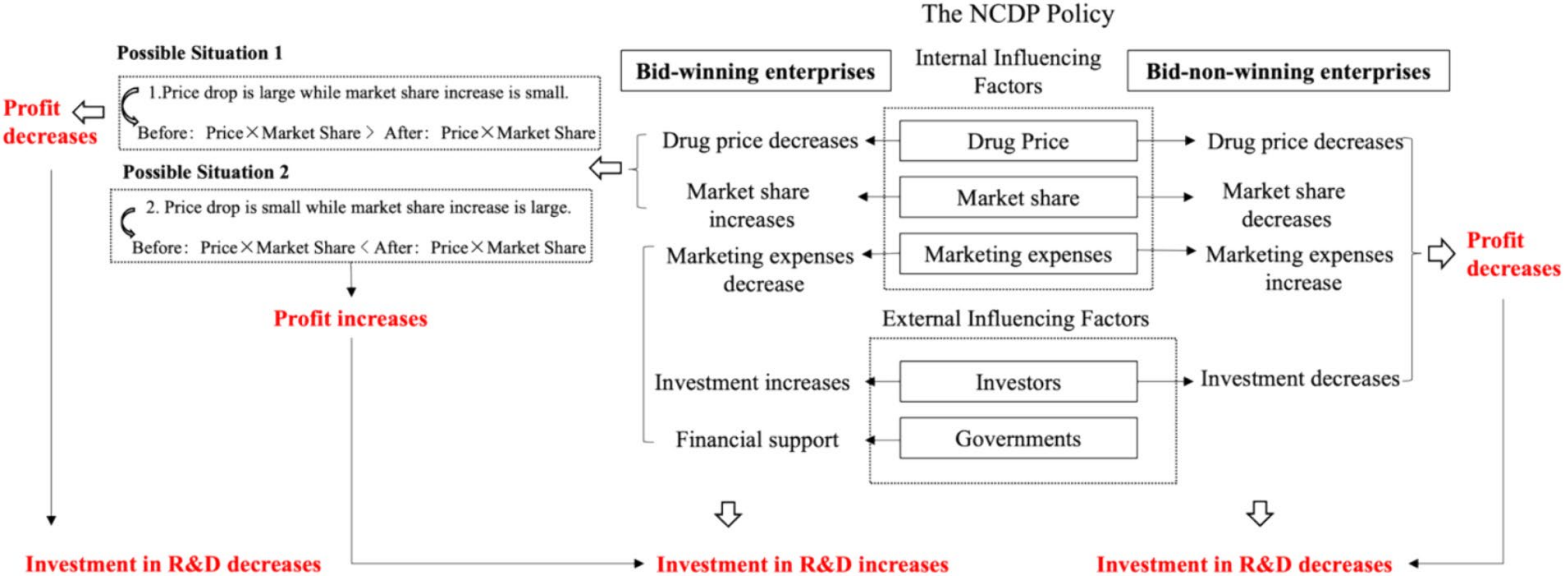
Impact pathway of the NCDP policy.

The impact of the NCDP policy on chemical pharmaceutical enterprises’ R&D investment can be divided into internal and external aspects (32, 33). The impact pathway is shown in Figure 1.

##### 2.1.1.1 Internal influence mechanism

Internal influence mechanism refers to the impact that the NCDP policy has on enterprises’ drug price, market share, and marketing expenses, which then affects their profits and R&D investment.

###### 2.1.1.1.1 Drug price and market share

To achieve the purpose of reducing drug prices, the NCDP policy guarantees bid-winning enterprises’ procurement volume and market share and ensures actual clinical utilization volume using administrative intervention. For bid-winning enterprises, whether profits would increase depends on the price reduction and market share increase. Therefore, policy effects vary from enterprise to enterprise, which is further analyzed in heterogeneity factors. For bid-non-winning enterprises, although the NCDP policy stipulates that 20 to 50% of public medical institutions’ utilization volume is reserved for bid-non-winning enterprises (34). In real situations, the procurement agreement does not specify the upper limit of the volume supplied by bid-winning enterprises. As a result, the real volume supplied by bid-winning enterprises often exceeds the agreed procurement quantity (35). The *Notice on the Implementation of National Centralized Drug Procurement* issued by Jiangxi Province on October 27, 2020, shows that in less than half a year since the implementation of the second round of NCDP, 26 of the 36 policy-related varieties have completed the agreed procurement volume, of which abiraterone tablet reached 2433.85% of the agreed volume (36). It is difficult for bid-non-winning enterprises to compete for the remaining market share, and the revenue of related drugs declines inevitably. In addition, the NCDP policy stipulates that bid-non-winning enterprises must also reduce price so that they can continue to participate in the procurement, further reducing product revenue space.

###### 2.1.1.1.2 Marketing expenses

On the one hand, research shows that marketing expenses would hinder the sustainable development of enterprises, occupy R&D investment, and weaken the core competitiveness of enterprises (37). However, the NCDP policy changes the traditional drug distribution chain, enabling bid-winning enterprises to sell drugs at the bid-winning price and fulfill the agreed procurement volume directly. This process helps save the cost of academic promotion, market information collection, and other marketing expenses (38, 39). On the other hand, the NCDP policy stipulates that the local medical insurance fund should pay not less than 30% of the procurement amount in advance to medical institutions and supervise medical institutions to pay enterprises on time (40). This helps reduce the financial pressure on bid-winning enterprises and encourages them to launch new R&D activities. By contrast, to make up for the lost medical institution share and maintain the original market share, bid-non-winning enterprises may try to open up out-of-hospital sales channels and compete for pharmaceutical retail market share, thus increasing marketing expenses (41).

##### 2.1.1.2 External influence mechanism

External influence mechanism refers to the impact that the NCDP policy has on the decision of external stakeholders, such as government and investors, which influences enterprises’ R&D investment indirectly.

###### 2.1.1.2.1 Government

The government’s support poses a positive impact on the R&D in the pharmaceutical industry (42, 43). The Chinese government actively promotes the implementation of the NCDP policy. To encourage the participation of enterprises, some local governments provide financial compensation to bid-winning enterprises. For example, bid-winning enterprises in Hangzhou, Shenzhen, and some other regions would be rewarded with 3% of the total procurement price for the year (44, 45).

###### 2.1.1.2.2 Investor

According to Signaling Theory, bid-winning enterprises can increase market share, which helps improve their reputation and popularity (46). This allows investors to easily identify promising enterprises, bridging the gap in information between investors and enterprises, easing financial constraints, and offering crucial funding for high-risk, high-cost, and long-term pharmaceutical innovations. On the contrary, failure to win the bid would directly lead to the decline of the enterprises’ stock price (47), resulting in investors’ lowered confidence and expectation for the enterprises’ future revenue. In addition, bid-non-winning enterprises tend to have lower attractiveness for talented people, indirectly leading to the decline of the enterprise’s R&D ability.

#### 2.1.2 Heterogeneity factors

The degree of impact of the NCDP policy also varies with the bid-winning enterprises’ bid success rate, drug price reduction, enterprise size, and other factors.

##### 2.1.2.1 Bid success rate

Bid success rate refers to the proportion of bid-winning drugs to all drugs involved in the NCDP for a single enterprise. If the bid success rate is low, it may cause serious losses to the enterprise’s profits. If the bid success rate is high, it reflects that the enterprise’s drugs have a greater competitive advantage. Compared to enterprises with low bid success rates, enterprises with high bid success rates are more likely to save marketing expenses, attract external investment, and obtain government’s financial support (48).

##### 2.1.2.2 Drug price reduction

After winning a bid, an enterprise can experience significant growth in market share and sales volume with a modest decrease in drug prices, which will lead to improved business performance in future. Coupled with other advantages brought by winning the bid, its profits will continue to grow, thus laying a financial foundation for R&D activities. However, according to the NCDP policy, the lower the bidding price, the more likely that the enterprise has the priority to choose a supply region and obtain a bigger market share (49). Therefore, under the pressure of obtaining high market share, enterprises may try to increase the chance of winning the bid by offering extremely low bidding prices. If the price reduction exceeds the enterprise’s affordability without the substantial growth of its market share, the enterprise’s profits would decline (22, 50), which directly affects the enterprise’s R&D enthusiasm.

##### 2.1.2.3 Enterprise size

Research shows that generally R&D investment is correlated with enterprise size positively (51, 52). This study anticipated that the NCDP policy had different impacts on the R&D investment of enterprises of different sizes. Enterprises of different sizes vary in R&D resources, R&D willingness, and risk tolerance, which results in their different policy sensitivity and the NCDP policy’s different incentive effect on them (53). Small- and medium-sized enterprises have limited start-up capital and lack experience, their R&D risk is relatively higher, and it is more difficult for them to bear the cost of failure. Before the promotion of the NCDP policy, most small- and medium-sized enterprises held a hesitant attitude toward the NCDP policy. By contrast, large enterprises with high market shares already have significant profits, well-established internal management systems, and access to ample financing options. They also possess the technical expertise to anticipate and manage R&D risks and are capable of shouldering substantial R&D costs. Faced with the opportunity of enterprise transformation provided by the NCDP policy, the greater the investment of large-scale enterprises, the more significant the incentive effect of the policy on the enterprise’s R&D (54).

### 2.2 Study hypothesis

Based on the above theoretical analysis, it can be inferred that, under the combined influence of internal and external factors, if drug price reduction can be realized with the maintenance of reasonable profits, the NCDP policy will contribute to the increase of bid-winning enterprises’ revenue. In addition, if the NCDP policy can reduce the marketing expenses of bid-winning enterprises and increase other income, the overall profits will increase, thus laying a good financial foundation for bid-winning enterprises to invest more in R&D. In addition, for enterprises with high bid success rate, small drug price reduction, and large size, they are less likely to suffer from the negative impact of unsuccessful bid and are more motivated in R&D, while bid-non-winning enterprises suffer more from financial negative impact, which may affect their R&D enthusiasm. Based on this, this study proposed the hypotheses H1, H1a, H1b, and H1c, as shown below:

*H_1_*: The NCDP policy promotes the R&D investment of bid-winning enterprises more than bid-non-winning enterprises.

*H_1a_*: The NCDP policy promotes the R&D investment of enterprises with high bid success rates more than enterprises with low bid success rates^1^.

*H_1b_*: The NCDP policy promotes the R&D investment of enterprises with small drug price reductions more than enterprises with large price reductions^2^.

*H_1c_*: The NCDP policy promotes the R&D investment of large-scale enterprises more than small- and medium-sized enterprises.

## 3 Study design

DID is widely used in the field of policy evaluation. It has three main advantages. First, it can overcome the reciprocal influence between the independent and dependent variables, avoiding endogeneity issues. Second, the method of introducing between-group dummy variables, time dummy variables, and their interaction terms into the econometric model is simple and easy to operate. Third, by regressing individual data using the DID model and judging the effectiveness of the policy through statistical significance, it can effectively overcome the problem of “spurious correlations” compared to other methods. Therefore, this quasi-natural experiment model can effectively conduct quantitative evaluations and has been widely applied in the field of public health policy evaluation in recent years (55). Some scholars have already used the DID model to empirically study changes in profitability, financial performance, and innovation performance of enterprises before and after the implementation of the NCDP policy (23, 56, 57).

### 3.1 Sample selection and data

The varieties involved in the first five rounds of NCDP are all chemical drugs. Therefore, according to *Shenwan Industry Classification Standards (2021 edition)*, the 153 A-share listed enterprises of the Shanghai Stock Exchange and Shenzhen Stock Exchange that belong to the chemical pharmaceutical subsector in the pharmaceutical and biological sector were selected as the sample, and the data from their annual reports from 2016 to 2022 were selected for analysis. To ensure the integrity and reliability of the sample data, the following enterprises were excluded: (1) enterprises with consecutive losses (marked with ST and *ST); (2) enterprises with missing data on the research variables form 2016 to 2022. As a result, 714 sample observations (balanced panel data) from 102 enterprises were retained. In addition, to avoid the influence of outliers, all continuous variables were winsorized by 1% above and below.

This study obtained the enterprises’ financial data for each year from the Wind database, obtained the bidding information for each enterprise from the public documents published on Sunshine Medical Procurement All-in-one, and obtained drug variety information, price reduction of bid-winning drugs, and other information from enterprises’ annual report and other public webpages.

### 3.2 Regression method

Taking the NCDP policy as a natural experiment, this study employs the DID model to estimate the impact of the policy on enterprises’ R&D investment. By controlling other factors, the DID model can examine whether there is a significant difference in R&D investment between the treatment group and the control group before and after policy implementation. The DID model was constructed as Equation 1:

(1) $$RDI_{i,t}=\beta_{0}+\beta_{1}DID_{i,t}+\alpha X_{i,t}+\gamma_{i}+\mu_{t}+\varepsilon_{i.t}$$

The dependent variable, $RDI_{\mathrm{it}}$ , indicates R&D investment. The subscript *i* indicates the *i*-th enterprise. The subscript *t* indicates the *t*-th year. The coefficient $\beta_{1}$ is the net effect of the NCDP policy on enterprises’ R&D investment. $X_{\mathrm{it}}$ is a set of control variables composed of other internal factors that may affect R&D investment. $\gamma_{i}$ is the individual fixed effect of each enterprise. $\mu_{t}$ is the time fixed effect, and $\varepsilon_{\mathrm{it}}$ is the random disturbance term.

### 3.3 Variables

#### 3.3.1 Dependent variable

The dependent variable was enterprises’ R&D investment, referring to the previous research (58, 59), which was measured by the ratio of R&D expenses to business income.

#### 3.3.2 Independent variable

The core independent variable was the NCDP policy, which is a policy dummy variable, set as *Treat*. Since enterprises affected by the NCDP policy are mainly chemical pharmaceutical enterprises, this study selected the treatment group and control group based on 102 A-share listed chemical pharmaceutical enterprises^3^. If the enterprise’s drugs involve varieties of the first five rounds of NCDP, it was classified into the treatment group, and *Treat* was assigned value 1. If the enterprise’s drugs do not involve varieties of the first five rounds of NCDP, it was classified into the control group, and *Treat* was assigned a value 0. Among the 102 sample enterprises, 37 belonged to the control group, and 65 belonged to the treatment group. Based on whether the enterprise won the bid, enterprises in the treatment group were divided into 43 bid-winning enterprises and 22 bid-non-winning enterprises.

The time dummy variable was the policy implementation, set as Post. In December 2018, the NCDP was first piloted in four municipalities and seven sub-provincial cities (thus, it is called the “4 + 7” pilot). In September 2019, the NCDP policy was expanded to the remaining 25 provincial administrative regions in the China (thus, it is called the “4 + 7” expansion). Taking into account the time-lag effect of policy, this study took 2019 as the start of policy implementation. If the sample enterprise was in the year before 2019, *Post* was assigned a value 0; otherwise, *Post* was assigned a value 1.

The policy net effect variable was equal to the interaction term Treat*Post (DID) between the policy dummy variable (*Treat*) and the time dummy variable (*Post*). Its coefficient was also the focus of this study and could reflect the net effect of the NCDP policy.

#### 3.3.3 Control variables

Referring to the previous research (60, 61), this study chose seven variables, including enterprise age (Age), financial leverage (Lev), and return on assets (ROA) as control variables to control the influence of endogenous factors such as capital structure on the results. A summary of abbreviations and definitions of variables is shown in Table 1.

**Table 1:** Summary of abbreviations and definitions of variables.

Type	Variable	Abbreviation	Definition
Dependent variable	R&D investment	RDI	R&D input/sales revenue
Independent variables	Treatment variable	Treat	Dummy variable, assigned as 1 if the enterprise’s drug involved in the first five rounds of the NCDP, and 0 otherwise
	Time variable	Post	Dummy variable, assigned as 1 if the enterprise was observed after 31 December 2018
	VBP policy	DID	Treat* Post
Control Variables	Enterprise age	Age	Time since the establishment of the enterprise
	Financial leverage	Lev	Debt/asset
	Return on sssets	ROA	Net income /total assets
	Total assset turnover	TAT	Net sales /total assets
	Liquidity ratio	LR	Current assets/ total assets
	Current ratio	CR	Current assets/ current liabilities
	Operating net cash flow	OCF	ln(operating net cash flow)

## 4 Results

### 4.1 Descriptive statistics

The mean, standard deviation, minimum, and maximum of all variables are shown in Table 2. The maximum, minimum, and mean of the dependent variable R&D Investment (RDI) were 30.207, 0.595, and 5.661%, respectively, indicating that there was a large gap in the R&D investment among the sample enterprises.

**Table 2:** Results of descriptive statistics.

Variable	*N*	Mean	Sd	Min	Max
RDI	714	6.430	5.661	0.595	30.207
Age	714	20.833	6.035	6	45
Lev	714	32.871	16.844	3.402	75.209
ROA	714	7.644	7.533	−17.181	26.466
TAT	714	0.577	0.268	0.158	1.743
LR	714	51.506	15.240	18.266	87.170
CR	714	2.704	2.122	0.572	11.834
OCF	714	17.459	5.721	0	22.488

The correlations among all variables are shown in Table 3. The correlation coefficients of all variables were less than 0.5, indicating that there was no strong correlation. The possibility of multicollinearity was preliminarily ruled out. Table 4 shows the results of the multicollinearity test. The maximum variance inflation factor (VIF) was 2.17 and the mean was 1.55, both were below 10. It indicated that there was no multicollinearity, and all the selected variables were suitable for further study.

**Table 3:** Correlation among variables.

Variable	RDI	DID	Age	Lev	ROA	TAT	LR	CR	CFO
RDI	1.000								
DID	0.086	1.000							
Age	−0.057	0.308	1.000						
Lev	−0.120	0.226	0.051	1.000					
ROA	−0.029	−0.204	−0.143	−0.374	1.000				
TAT	−0.240	0.046	−0.142	0.017	0.438	1.000			
LR	−0.042	−0.014	0.005	−0.195	0.245	0.339	1.000		
CR	0.156	−0.116	0.040	−0.625	0.249	−0.078	0.442	1.000	
OCF	−0.015	−0.016	−0.007	−0.144	0.281	0.149	−0.076	0.055	1.000

**Table 4:** Multicollinearity test.

Variable	VIF	1/VIF
CR	2.17	0.4600
Lev	1.89	0.5281
ROA	1.64	0.6092
TAT	1.64	0.6223
LR	1.60	0.6269
DID	1.20	0.8299
Age	1.15	0.8726
OCF	1.13	0.8818
Mean VIF	1.55	

### 4.2 DID regression

As analyzed above, the NCDP policy has different impacts on bid-winning and bid-non-winning enterprises. Therefore, this study conducted DID regression on bid-winning and bid-non-winning enterprises, respectively. Enterprises in the treatment group were classified according to the experience of Fan Ziying (2022) (62) and Hope O.K. (63) and then performed regression with the control group to reduce the interference of the grouping and enhance the comparability of results.

#### 4.2.1 Results of DID regression

The results of DID regression on bid-winning enterprises are shown in Table 5. In particular, column (1) is the regression results on bid-winning enterprises without adding control variables. The DID coefficient is 1.815, significant at the 1% level. Column (2) is the regression results on bid-winning enterprises with control variables. The DID coefficient increases to 1.834, still significant at the 1% level. Column (3) is the regression results on bid-non-winning enterprises without adding control variables. The DID coefficient is 1.069, significant at the 5% level. Column (4) is the regression results on bid-non-winning enterprises with control variables. The DID coefficient is 1.310, significant at the 5% level. These results support H_1_ that the NCDP policy promotes the R&D investment of bid-winning enterprises more than bid-non-winning enterprises.

**Table 5:** DID regression results.

Variable	(1)	(2)	(3)	(4)
	RDI	RDI	RDI	RDI
DID	1.815^***^ (0.648)	1.834^***^ (0.615)	1.069^**^ (0.526)	1.310^**^ (0.572)
Age		0.121 (0.093)		0.069 (0.086)
Lev		−0.046 (0.028)		−0.024 (0.014)
ROA		−0.198^***^ (0.064)		−0.090 (0.050)
TAT		0.725 (1.578)		−1.420 (1.057)
LR		−0.042 (0.025)		−0.019 (0.020)
CR		0.059 (0.119)		0.048 (0.075)
CFO		−0.000 (0.028)		−0.049^*^ (0.027)
_cons	5.796^***^ (0.309)	6.432^***^ (1.070)	5.796^***^ (0.309)	8.382^***^ (2.650)
Firm FE	YES	YES	YES	YES
Year FE	YES	YES	YES	YES
*N*	560	560	413	413
R^2^	0.143	0.268	0.094	0.233

Standard errors in parentheses; *p < 0.1, **p < 0.05, and ***p < 0.01.

#### 4.2.2 Heterogeneity analysis of BID-winning enterprises

As analyzed above, the R&D investment of bid-winning enterprises varies according to the bid success rate, the drug price reduction of bid-winning drugs, and the enterprise size. Therefore, this study conducted a heterogeneity analysis based on these factors.

##### 4.2.2.1 Bid success rate

This study took the ratio of the number of bid-winning drugs in the first five rounds of NCDP to the number of varieties involved in the first five rounds of NCDP as the enterprise’s bid success rate. The average success rate of all bid-winning enterprises was used as the dividing line. Those above the line were classified as enterprises with high bid success rates, and those below the line were classified as enterprises with low bid success rates. Regression analysis was performed on the two types of enterprises separately, and the results are shown in Column (1) and Column (2) of Table 6. Column (1) is the regression results of enterprises of high bid success rate with control variables. The DID coefficient is 2.535, significant at the 1% level. Column (2) is the regression results of enterprises of low bid success rate with control variables. The DID coefficient is 1.217, significant at the 10% level. These results indicate that the NCDP policy promotes the R&D investment of enterprises with high bid success rates more than enterprises with low bid success rates, which supports H_1a_.

**Table 6:** Results of heterogeneity analysis.

Variables	(1)	(2)	(3)	(4)	(5)	(6)
	RDI	RDI	RDI	RDI	RDI	RDI
DID	2.535^***^ (0.874)	1.217^*^ (0.645)	0.545 (0.717)	3.400^***^ (0.809)	2.000^***^ (0.502)	1.536 (2.105)
Age	0.069 (0.106)	0.141^*^(0.076)	0.132 (0.923)	0.100 (0.090)	0.147^*^(0.085)	0.061 (0.114)
Lev	−0.054^**^ (0.026)	−0.022 (0.025)	−0.044^*^ (0.024)	−0.043 (0.031)	−0.072^***^ (0.021)	0.003 (0.114)
ROA	−0.172^**^ (0.810)	−0.153^***^ (0.049)	−0.141^***^ (0.051)	−0.195^**^ (0.084)	−0.155^**^ (0.069)	−0.179^**^ (0.069)
TAT	−0.946 (2.061)	0.741 (0.980)	0.647 (1.505)	0.528 (1.719)	0.887 (1.642)	−0.939 (1.444)
LR	−0.067^**^ (0.031)	−0.010 (0.020)	−0.036 (0.029)	−0.028 (0.023)	−0.071^***^ (0.024)	0.016 (0.025)
CR	0.095 (0.133)	0.019 (0.068)	0.132 (0.098)	−0.037 (0.097)	0.131 (0.092)	−0.064 (0.117)
CFO	0.000 (0.031)	−0.002 (0.023)	−0.016 (0.027)	−0.001 (0.016)	−0.019 (0.026)	0.016 (0.032)
_cons	12.149^***^ (2.658)	5.862^***^ (1.663)	7.936^***^ (1.973)	9.165^***^ (2.563)	10.095^***^ (2.122)	7.214^**^ (2.774)
Firm FE	YES	YES	YES	YES	YES	YES
Year FE	YES	YES	YES	YES	YES	YES
*N*	406	413	420	399	504	315
R^2^	0.290	0.222	0.199	0.349	0.339	0.194

Standard errors in parentheses; *p < 0.1, **p < 0.05, and ***p < 0.01.

##### 4.2.2.2 Price reduction of bid-winning drugs

This study calculated the average price reduction of bid-winning drugs of each enterprise and took the average price reduction of all bid-winning enterprises as the dividing line. Those above the line were classified as enterprises with large price reductions, and those below the line were classified as enterprises with small price reductions. Regression analysis was performed on the two types of enterprises separately, and the results are shown in Column (3) and Column (4) of Table 6. Column (3) is the regression results of enterprises with large price reductions with control variables. The DID coefficient is not significant, indicating that the NCDP policy did not have a significant impact on the R&D investment of enterprises with large price reductions. Column (4) is the regression results of enterprises with small price reductions with control variables. The DID coefficient is 3.400, significant at the 1% level, indicating that the NCDP policy increased the R&D investment of enterprises with small price reductions by 3.400%, which supports H_1b_.

##### 4.2.2.3 Enterprise size

To investigate the relationship between R&D investment and enterprise size under the NCDP policy, this study divided bid-winning enterprises into large-scale enterprises and small- and medium-sized enterprises according to *Statistical Methods for the Classification of Large, Medium, Small and Micro Enterprises (2017)* issued by the National Bureau of Statistics and performed regression analysis separately. The results are shown in Column (5) and Column (6) of Table 6. Column (5) is the regression results of large-scale enterprises. The DID coefficient is 2.000, significant at the 1% level. Column (5) is the regression results of small- and medium-sized enterprises. The DID coefficient is not significant, indicating that the NCDP policy promotes the R&D investment of large-scale bid-winning enterprises by 2.000% but produces no significant effect on small- and medium-sized bid-winning enterprises, which supports H_1c_.

### 4.3 Robustness tests

#### 4.3.1 Parallel trend test

**Figure 2:**
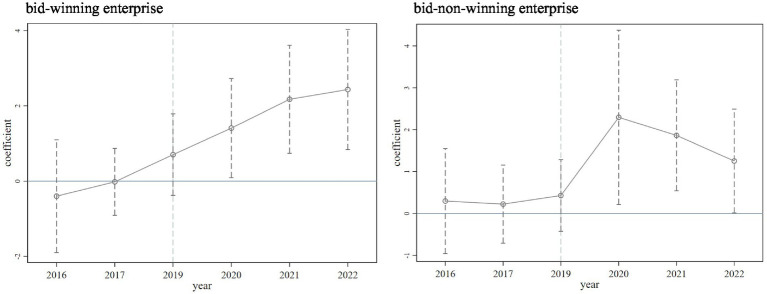
Parallel trend test (with control variables).

To use the DID model, the assumption that there is a common trend between the treatment and control groups must be valid. In other words, before the implementation of the NCDP policy, the trends in overall R&D investment of the enterprises in the treatment and control groups must not differ systematically over time. The results of the parallel trend test are shown in Figure 2. As can be seen, before the time point of the NCDP policy, the annual regression coefficients of the bid-winning and bid-non-winning enterprises were close to 0, and the 95% confidence interval covered the 0 points, which was not significant, indicating that the samples passed the parallel trend test. After the time point of the NCDP policy, the confidence interval of the regression coefficient of bid-winning enterprises was different from 0 and gradually moved away from 0. In contrast, the confidence interval of the regression coefficient of bid-non-winning enterprises gradually approached 0. This suggests that with the continuous improvement of the NCDP policy, the R&D promotion effect of the policy on bid-winning enterprises increased over time, while the increase in R&D investment by bid-non-winning enterprises decreased over time.

#### 4.3.2 Placebo test

**Figure 3:**
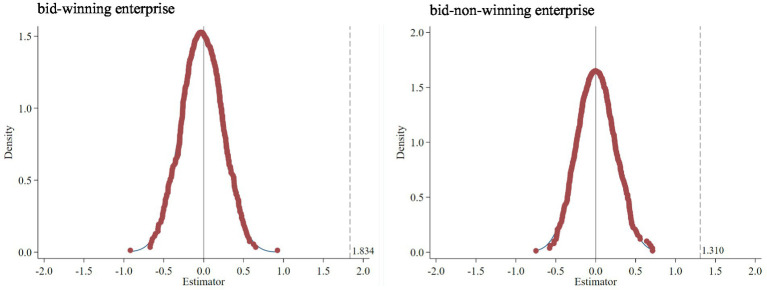
Placebo test.

The regression results of this study were produced under the double fixed effect of enterprise and year, but there may exist the influence of random factors or omitted variables that were difficult to observe. Therefore, referring to the ideas of La Ferrara (64) and Lu Yue (65), this study tested whether indirect unobservable factors would affect the benchmark regression results. This study randomly generated treatment groups for the sample data, randomly selected a time for each treatment group as the policy implementation time point, generated “pseudo-dummy variables,” and performed regression according to the aforementioned main model. This process was repeated 500 times, resulting in 500 random sampling regressions. Figure 3 shows the distribution of the 500 estimated coefficients of the “pseudo-dummy variables.” The coefficients generally conformed to a normal distribution with a mean of 0 and were far from the true DID model estimate (1.834 of bid-winning enterprises and 1.310 of bid-non-winning enterprises). This indicated that there was no obvious problem of omitted variables in the benchmark regression model, and other factors that may lead to bias had little impact on the results. Therefore, this study passed the placebo test, and the previous conclusions were robust.

## 5 Conclusion and discussion

This study used the two-way fixed effects DID model to evaluate the impact of the NCDP policy on the R&D investment of listed pharmaceutical enterprises in China and concluded as follows:

It could be seen from the regression results that the NCDP policy had a positive promotion effect on the R&D investment of chemical pharmaceutical enterprises, and the promotion effect on bid-winning enterprises was more obvious than on bid-non-winning enterprises. Research hypothesis H1 was established. This result was consistent with the findings from previous studies (23, 66). The NCDP policy eliminated excessive costs in drug circulation, prompting companies to shift from competing based on sales channels to focusing on quality and pricing. This shift has incentivized enterprises to invest more in technological innovation. In addition, bid-winning enterprises could obtain a stable sales volume, repayment guarantee, and government funding, having a good expectation of market share and profit level for the next year. While bid-non-winning enterprises would be subject to the double pressure of market shrinkage and declined profit level, which affected their R&D investment. Therefore, bid-winning enterprises were more willing and able to increase investment in R&D.

The results of heterogeneity analysis indicated that, among bid-winning enterprises, the NCDP policy had a more obvious promotion effect on large-scale enterprises with high bid success rates and small price reductions. Research hypotheses H1_a_, H1_b_, and H1_c_ were established. Of all the factors, drug price reduction was the most influential one; enterprises with small drug price reduction had the highest promotion level of R&D investment. This finding further validates and supplements previous studies (53, 56), indicating that high price reductions would lead to decreases in the net profits of bid-winning enterprises, thereby reducing their willingness to invest in R&D. Additionally, to cope with pricing pressures, enterprises may allocate more resources to reduce costs and improve efficiency, impacting enterprises’ R&D process. Because the increase in sales volume was enough to resist the negative effect caused by the decrease in drug prices, the overall profits of the enterprise could be increased, thus laying a good financial foundation for the R&D. The enterprise was able to develop new drugs continuously, improving its competitiveness in the subsequent NCDP. By contrast, a large drug price reduction would result in a serious loss of profits and reduce the enterprise’s willingness to invest in R&D. At the same time, to cope with the pressure of price reduction, the enterprise would invest more resources in reducing costs and improving efficiency, which would affect the R&D process of the enterprise.

There are several policy insights from this study. First, as an indirect innovation incentive policy, the NCDP policy reduces the expenditure on the circulation, sales, and promotion of bid-winning drugs. Therefore, it can significantly encourage bid-winning enterprises to invest more in R&D. Meanwhile, the NCDP policy can urge bid-non-winning enterprises to speed up the process of drug innovation, guiding them to differentiated innovation paths. Therefore, the government should continue to implement the NCDP policy, realizing the overall transformation and upgrading of innovation in the pharmaceutical industry. Second, the NCDP policy still has some deficiencies in promoting the R&D of enterprises. Low bid success rates and large drug price reductions may affect the R&D enthusiasm of enterprises, especially small- and medium-sized enterprises. Therefore, the bidding rules and drug price reduction mechanisms of the NCDP policy still need to be further optimized. This study makes the following suggestions:

First, optimize the drug price reduction mechanism. The NCDP policy is implemented under the leadership of the government and uses huge market share to encourage enterprises to voluntarily disclose price information, thereby seeking a better balance between enterprises’ interests and people’s livelihood. To master the “balance point,” the government needs to take into account the reasonable profits of enterprises while reducing the inflated prices of drugs. Therefore, the government should optimize the drug price reduction mechanism and the market distribution mechanism to avoid excessive drug reduction caused by malicious competition among enterprises (67).

For one thing, for drugs with excessive price reduction or extremely low prices, establish a low-price identification mechanism. Identification methods can refer to the following two: The first is to compare with the international reference prices. Focus on the lowest prices in neighboring countries or regions such as Japan, South Korea, and Hong Kong. If the bidding price is significantly lower than the lowest price, the bidding price can be considered too low (68). The second is to set an abnormal price reduction threshold (69). Calculate the abnormal price reduction threshold based on the reduction of each valid bid. If the price reduction exceeds this threshold, the bidding price can be considered too low. Once the medical insurance department deems that the bidding price is too low, the bidding enterprise should issue a cost calculation and other materials to explain. If the reason is reasonable, the bid will be accepted. Otherwise, the bid will be rejected, and the bid winner will be selected from other bidding enterprises according to the NCDP rules.

For another, introduce a two-way selection mechanism between medical institutions and supply enterprises (70). By controlling the independent right of choice of bid-winning enterprises, the NCDP policy can prevent enterprises from over-bidding to obtain the supply regions they want. In particular, after the enterprise with the lowest bidding price selects a region, other enterprises select regions in order from low-to-high price. When selecting, each enterprise needs to match the selection of the region. If the matching is unsuccessful, the enterprise will continue to select until mutual matching is successful. In addition, the NCDP policy should also consider the R&D costs of drugs. Pharmacoeconomic evaluation can be gradually integrated into the NCDP policy to maximize the cost-effectiveness of the procurement and form healthy competition among enterprises (71).

Second, optimize the number of bid-winning enterprises. The increase in bid success rate is conducive to promoting drug R&D. When formulating rules for the NCDP policy, the government may consider increasing the number of bid-winning enterprises (72). While maintaining quality control, it is encouraged to select multiple enterprises of the same variety to participate. At the same time, enterprises should be required to strengthen confidentiality measures to prevent speculative activities (73). This approach would not only stimulate healthy competition among enterprises but also avoid exclusive drug monopoly and reduce the negative impact of local drug shortages in case the bid-winning enterprises cut off supply.

Third, pay attention to small- and medium-sized enterprises. For innovative small- and medium-sized enterprises, increase the intensity of the NCDP policy. By increasing procurement share, shortening payment deadlines, and providing more advance payments, funds can be promptly transferred to small- and medium-sized enterprises to address cash flow shortages and reduce transaction costs. In addition, the government can consider setting up a special compensation fund for R&D activities to provide appropriate financial compensation, helping small- and medium-sized enterprises solve financing problems and achieve corporate transformation.

Compared with previous studies, this study offers several innovative points. First, it provides a novel research perspective. Current studies on the NCDP policy mainly focus on the policy’s effects on drug costs and usage. Few studies have explored the impact on enterprises’ R&D development. This study examines the impact on enterprises’ R&D development from the stakeholder perspective, adding new content to the research field. Second, our study introduces an innovative research perspective. While existing literature does not classify pharmaceutical enterprises in detail, this study divides enterprises into bid-winning and bid-non-winning ones, providing a more objective observation of the policy’s impact. Third, our study presents innovative research conclusion. This study utilizes the DID model to alleviate endogeneity issues in general regression analysis, enhancing the credibility of the research conclusion. The results confirm that the NCDP policy has a positive effect on the innovation of pharmaceutical enterprises, with the intervention effect being greater for bid-winning enterprises than for bid-non-winning enterprises. Additionally, this study finds that price reductions, bid success rate, and enterprise size are factors that influence the policy intervention effect. Targeted strategies are proposed based on the issues identified in the study, contributing to the improvement of the NCDP policy.

Our study also has several limitations. First, to reduce the regression bias, this study chose to perform regression after eliminating missing values. Therefore, the sample size of the study was relatively small and could not be completely scientific at the data level. Second, due to the difficulty in obtaining market share change and operating profit data of a single drug, there were no relevant data available in public databases; therefore, this study was unable to conduct an empirical analysis on these two factors. Third, this study failed to demonstrate the direction and efficiency of the R&D investment. In terms of R&D investment direction, this study was unable to explain whether the NCDP policy encouraged enterprises to invest more in innovative drugs or first generic drugs with high technical barriers. In terms of R&D investment efficiency, this study was unable to explain whether the number of approved new drugs increased under the premise of the same R&D investment. Since the R&D of innovative drugs requires long-term investment, it is difficult to see R&D results in the short term. It is hoped that in future, data with a longer time range and more dimensions (such as the number of patent applications and the number of innovative drugs/first generic drugs approved) can be collected to further evaluate the impact of the NCDP policy on the R&D investment of chemical pharmaceutical enterprises.

## Data availability statement

The original contributions presented in the study are included in the article/supplementary material, further inquiries can be directed to the corresponding authors.

## Author contributions

JL: Conceptualization, Data curation, Investigation, Methodology, Writing – original draft, Writing – review & editing. XZ: Conceptualization, Data curation, Investigation, Methodology, Writing – original draft, Writing – review & editing. RW: Conceptualization, Data curation, Investigation, Methodology, Writing – original draft, Writing – review & editing. KC: Writing – original draft. LW: Writing – original draft. XR: Conceptualization, Methodology, Writing – review & editing. JD: Conceptualization, Writing – review & editing, Supervision. WL: Conceptualization, Writing – review & editing, Supervision.

## Footnotes

^1^Taking the average bid success rate as the dividing line, those above the average level are classified as bid-winning enterprises with high bid success rate, and vice versa are classified as bid-winning enterprises with low bid success rate.

^2^Taking the average price reduction as the dividing line, those above the average level are classified as bid-winning enterprises with large price reduction, and vice versa are classified as bid-winning enterprises with small price reduction.

^3^In view of back door listing and affiliation, the names and stock codes of the listed chemical pharmaceutical had been compared one by one, and the backdoor and affiliated enterprises were unified into the same enterprise.
